# Architecture Exploration of a Backprojection Algorithm for Real-Time Video SAR

**DOI:** 10.3390/s21248258

**Published:** 2021-12-10

**Authors:** Seokwon Lee, Inmo Ban, Myeongjin Lee, Yunho Jung, Wookyung Lee

**Affiliations:** 1 Department of Smart Air Mobility, Korea Aerospace University, Goyang 10540, Korea; swlee2542@kau.kr (S.L.); yjung@kau.ac.kr (Y.J.); 2 School of Electronics and Information Engineering, Korea Aerospace University, Goyang 10540, Korea; babe4519@kau.kr (I.B.); wklee@kau.ac.kr (W.L.)

**Keywords:** synthetic aperture radar, SAR, video SAR, backprojection, sub-aperture processing, overlapped aperture mode

## Abstract

This paper explores novel architectures for fast backprojection based video synthetic aperture radar (BP-VISAR) with multiple GPUs. The video SAR frame rate is analyzed for non-overlapped and overlapped aperture modes. For the parallelization of the backprojection process, a processing data unit is defined as the phase history data or range profile data from partial synthetic-apertures divided from the full resolution target data. Considering whether full-aperture processing is performed and range compression or backprojection are parallelized on a GPU basis, we propose six distinct architectures, each having a single-stream pipeline with a single GPU. The performance of these architectures is evaluated in both non-overlapped and overlapped modes. The efficiency of the BP-VISAR architecture with sub-aperture processing in the overlapped mode is accelerated further by filling the processing gap from the idling GPU resources with multi-stream based backprojection on multiple GPUs. The frame rate of the proposed BP-VISAR architecture with sub-aperture processing is scalable with the number of GPU devices for large pixel resolution. It can generate 4096 × 4096 video SAR frames of 0.5 m cross-range resolution in 23.0 Hz on a single GPU and 73.5 Hz on quad GPUs.

## 1. Introduction

Synthetic aperture radar (SAR) is an active sensing technology that can operate in all weather and in day or night air-to-ground surveillance. SAR produces rectilinear images by reconstructing the reflected radio-frequency (RF) signal from stationary objects on the ground. The high-resolution SAR images can be produced in range and cross-range directions with resolutions determined by the bandwidth of transmitted pulses and the Doppler induced by the platform’s motion. Recent applications include the detection of temporal changes of target areas and monitoring of moving targets. Thus, the concept of video SAR arises to fulfill the increasing demand for higher spatial and temporal resolutions, especially of moving ground targets. Naturally, this involves a heavy computational burden caused by increasing the data size and latency for multi-frame SAR image generation.

In general, SAR image formation methods are categorized into time-domain and frequency-domain algorithm groups. The use of matched filters in both range and azimuth directions is likely to generate SAR images with a maximum signal-to-noise ratio. However, the computational complexity increases in proportion to $O(N^{4})$ where *N* is the number of sampling points and makes it difficult to apply for fast processing in practical applications [1]. Convolution backprojection (CBP) [2] is an alternative to the matched filter with reduced computational complexity. By precomputing the pulse compression, one can interpolate the range to assess the contributions of every pulse to each pixel to achieve a complexity proportional to $O(N^{3})$. However, CBP is still computationally burdensome, even for moderate-size target scenes and is considered unsuitable for real-time SAR for wide-area scenes. Frequency-domain algorithms resample the collected phase history into a rectangular raster after preprocessing and enable bulk operations using fast Fourier transform (FFT) to maximize computational efficiency. These algorithms store a chunk of data collected from the maximum scene size to process it in a frequency transform domain at the expense of marginal defocus [3,4,5].

Conventional SAR image formation for real-time processing has long been researched in terms of computational efficiencies and hardware accelerations. Still, their performances are limited by the increased scene sizes, particularly in a video SAR mode, where the amount of incoming raw data increases rapidly with a higher frame rate. In particular, the requirement for a sub-meter resolution level makes it difficult to implement in real-time. In addition, ground moving target detection is employed, but there is no general rule that can accommodate ground targets moving in arbitrary directions and velocities. In general, SAR images do not allow real-time tracking of moving objects or rapid change detection because they are not continuously produced in streams like SAR video frames.

Recently, increased attention has focused on the video SAR system, which continuously senses a region of interest (ROI), while the moving radar platform operates either in flying by or circling the ROI in spotlight SAR modes [6,7,8,9]. Video SAR sequences are constructed by placing video SAR frames generated in consecutive time order. In principle, the signal processing for video SAR shares the same algorithm with the conventional SAR image generation, but the synthetic aperture must be configured to adapt for multiple observations in consecutive time sequences. The video frame rate needs to be high enough to allow applications to visually track moving objects through a video SAR frame sequence, extracting information such as the velocities, moving tracks, types of objects, etc. In video SAR, it is highly challenging to handle the computational loads of generating video SAR frames in real time while observing dynamic scenes in designated configurations. Real-time video SAR processing is essential for day-and-night surveillance on collaborative video SAR sensor networks, which is another type of Internet of Things (IoT) [10,11].

Recent studies on video SAR have focused on video SAR simulation [6,7,12,13], frame rate analysis [12,13,14], and field experiments [9,15,16,17]. All these studies construct a synthetic aperture for a video SAR frame in non-overlapped or overlapped aperture modes along the flight path of the platform. For the fast update of video SAR frames, it is generally preferred to construct a synthetic aperture in the overlapped mode.

In the non-overlapped mode, the computational load of video SAR frame generation is proportional to the frame rate. Although the computational load also increases proportionally to the frame rate in the overlapped mode, its performance depends on the configuration of the SAR frame generation. Frequency-domain SAR generation algorithms achieve computational efficiency through bulk operations using FFT. However, these algorithms cannot remove the redundant processes for phase history data supporting synthetic apertures for neighboring video SAR frames, as bulk operations constrain their design variation. The backprojection algorithm (BPA) can form video SAR frames of lower resolution on a pulse or a group of pulses basis every time phase history data is collected. Therefore, in a BPA, we can partition the phase history data collected from a full-synthetic-aperture scene into smaller units. By defining a sub-synthetic-aperture as a group of smaller pulses, we can parallelize the backprojection processes for sub-synthetic-apertures and merge their processing outputs into the full-aperture video frames.

The key advantages of the BPA are as follows. It can form SAR images with lower resolution in a pulse or a group of pulses every time phase history data is collected and progressively improve the resolution of the SAR images by superposing the images of lower resolution on the recently formed SAR images. In addition, BPA enables image formation on arbitrary imaging grids. When receive and transmit antennas are separated, as in passive or bistatic SAR configuration, the platform motion should be compensated with high precision and it is preferred to divide processing data into multiple blocks. Then, parallel BPA processing can be easily adopted to accelerate for real-time processing and SAR image generation [18,19,20]. This advantage of BPA has motivated many studies that accelerate the BPA in recent decades. Some approaches modified the architecture of BPA by spatially subdividing the computational load into multi-stage fast backprojection (FBP) and digital spotlighting (DS) algorithms [21]. Other accelerations are achieved by porting the computationally intensive part of the BPA into massively parallel devices such as general-purpose graphics processing unit (GPGPU) [22,23,24,25,26,27] and field-programmable gate array (FPGA) [28,29,30]. Currently, the image formation speed of these acceleration studies is not keeping up with that of frequency-domain algorithms such as polar format algorithm (PFA) under the same computing environment conditions. However, with the advent of multi-processing hardware technology, we argue that BPA is a candidate for real-time video SAR due to its parallelization characteristics and sub-aperture processing capability.

In this study, we explored the architecture of BPA-based video SAR (BP-VISAR) for multiple GPU hardware. For a scalable and high frame rate, we analyzed the video SAR frame rate for non-overlapped and overlapped modes. We designed BPA-based video SAR algorithms with full- and sub-aperture processing on the host CPU as a reference. After profiling the computational complexity of the BP-VISAR, we partitioned it into multi-stage sub-processes.

By allocating computational resources of GPU devices and enabling the sub-aperture based processing and buffering for the parallelization of the sub-processes, we propose architectures for BP-VISAR with full- and sub-aperture processing. In this paper, we first present three architectures of BP-VISAR with full-aperture processing for a single GPU system and evaluate their performance for a single video SAR stream. Second, we propose three architectures of BP-VISAR with sub-aperture processing for a single GPU system and evaluate their performances for a single video SAR stream. Third, we propose an N-way concurrent architecture of BP-VISAR for parallelization of multiple streams for BP-VISAR frame generation on a single GPU and evaluate its performance. Finally, we propose a multi-GPU based N-way concurrent architecture of BP-VISAR for further acceleration with scalable computational resources.

The contribution of this study is three-fold. First, the relationship between the video SAR frame rate and the radar system parameters is analyzed to help design the video SAR system in non-overlapped and overlapped synthetic aperture modes. Second, parallel pipelined architectures are proposed to handle the phase history data and the range profile data on a sub-synthetic-aperture basis and remove redundant computation in overlapped synthetic aperture modes. Finally, the proposed multi-stream BP-VISAR architecture on multiple GPUs is shown to generate video SAR frames in real-time for streaming phase history data that is scalable in the output frame rate with the number of GPU devices for frames of high pixel resolution.

This paper is organized as follows. Section 2 analyzes the relationship between the video SAR frame rate and the SAR system parameters. Section 3 presents architectures for backprojection based video SAR for full- and sub-aperture processing and multi-stream and multi-GPU extended architectures. Section 4 presents the experimental results of the proposed BP-VISAR architectures and finally, Section 5 gives our conclusions.

## 2. Frame Rate Analysis for Video SAR

Video SAR has been devised for onboard aircraft [6,7,9,15], drone [14,17] and satellites [12,13,16] platforms, and their operational modes are used for spot and tracking modes in circular [6,7,9,14,15,17] and fly-by paths [12,13,16].

For the generation of seamless SAR frames upon the streaming phase histories of the moving targets, the video SAR system needs to meet strict processing time constraints that are not plausible with the conventional SAR processors. These constraints become a critical performance measure that limits the frame rate of a video SAR system. The design of the video SAR system must take into account that the processing time varies according to the SAR operating mode, a function of antenna steering, the overlapping ratio of the aperture length, the bandwidth, PRF, and swath width. The difficulty is escalated when the time-varying properties of the incoming signals from the moving targets demand reduction of each frame’s synthetic aperture length.

**Figure 1:**
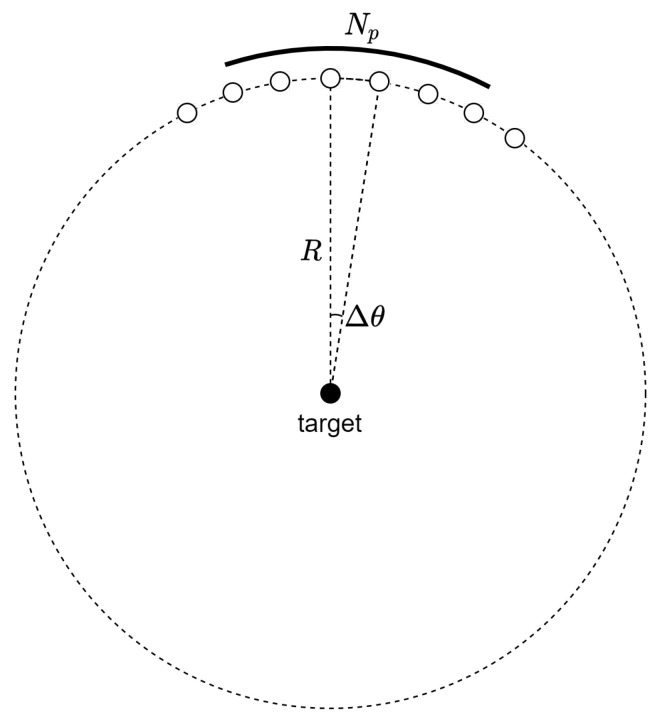
Geometry for circular video SAR.

A video SAR system with a circular path has the geometry shown in Figure 1. The azimuth step size Δθ is calculated as follows.

(1) $$\Delta \theta =\frac{V_{s}}{R\cdot f_{PRF}},$$

where $f_{PRF}$, $V_{s}$, and *R* represent the pulse repetition frequency (PRF), platform velocity, and operating range, respectively.

If a synthetic aperture for a single video SAR frame is constructed with $N_{p}$ pulses, the synthetic aperture angle is given as follows.

(2) $$\theta_{a}=N_{p}\Delta \theta .$$

Then, the corresponding synthetic aperture time is as follows.

(3) $$T_{a}=\frac{N_{p}}{f_{PRF}}.$$

**Figure 2:**
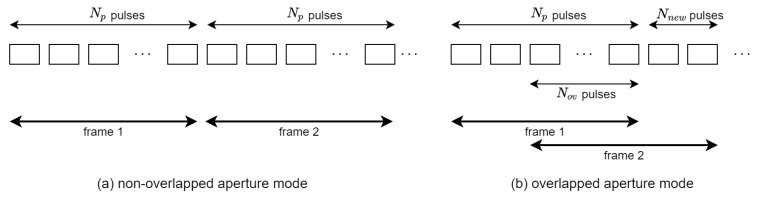
Non-overlapped and overlapped apertures for video SAR frame construction.

Video SAR frames can be constructed either in non-overlapped or in overlapped modes, as in Figure 2. The non-overlapped mode is a special case of the overlapped mode where the overlap ratio is zero. As in the previous works on video SAR frame rate analysis [12,13,14], the frame rate for the overlapped mode is as follows.

(4) $$\begin{array}{ccc} \gamma & = & \frac{V_{s}}{R\Delta \theta (N_{p}-N_{ov})} \end{array}$$

(5) $$\begin{array}{ccc} & = & \frac{f_{PRF}}{N_{p}-N_{ov}}, \end{array}$$

where $N_{ov}$ represents the number of overlapped pulses used for the generation of neighboring video SAR frames.

The cross-range resolution is not affected by the overlap ratio nor the frame rate and is given as follows.

(6) $$\rho_{a}=\frac{\lambda }{2\theta_{a}}=\frac{\lambda R}{V_{s}}\cdot \frac{f_{PRF}}{N_{p}},$$

where λ represents the RF wavelength of the transmitted pulses.

## 3. Architecture Exploration for Backprojection Based Video SAR

### 3.1. Backprojection Based Video SAR

A video SAR system transmits pulses at regular intervals and samples the reflected pulses from the target area in a phase history format. Phase history represents streaming data that inherits a group of pulse signatures that correspond to the SAR frame at a specific moment. Because the cross-range resolution improves with the increasing length of the synthetic aperture, BP-VISAR sub-frames generated by a small group of pulses can be superposed to construct a full BP-VISAR frame having improved cross-range resolution.

From the nature of the backprojection algorithm, the computation for BP-VISAR frame formation can be easily parallelized. The group of pulses for a full-synthetic-aperture is partitioned into smaller groups for sub-synthetic-aperture processing. Each smaller group can be used to construct a group of BP-VISAR frames with low cross-range resolutions. This sub-group processing is performed independently and can be parallelized. Unlike the non-overlapped mode in Figure 2a, in the overlapped mode in Figure 2b, multiple sub-aperture pulses in partial overlap intervals are reused to construct neighboring BP-VISAR frames. This enables the multiple BP-VISAR frames with a low cross-range resolution to be superposed into BP-VISAR frames with a higher resolution. This helps avoid redundant processing for the overlapped domain data, and the frame rate can be increased with reduced computational load in the overlapped BP-VISAR mode.

**Figure 3:**
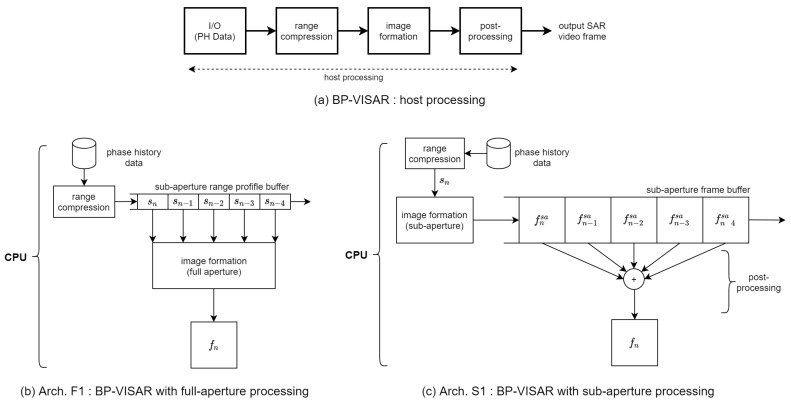
BP-VISAR baseline architectures.

The processing stages to implement BP-VISAR consist of I/O, range compression (RC), image formation (IF), and post-processing (PP), as illustrated in Figure 3a. It is assumed that $N_{p}$ pulses are employed to construct a single BP-VISAR frame. The *n*th pulse is transmitted at $\tau_{n}$. The received signal is transformed into a frequency domain after A/D conversion, and the *k*th frequency sample for the *n*th reflected pulse is denoted by $S(f_{k},\tau_{n})$.

Given phase history data $S(f_{k},\tau_{n})$, the range profile at range bin *m* for a received pulse at $\tau_{n}$ is given as follows.

(7) $$s\left(m,\tau_{n}\right)=\sum_{k=1}^{K}S\left(f_{k},\tau_{n}\right)\exp\left(\frac{j4\pi f_{k}\Delta R\left(m,\tau_{n}\right)}{c}\right),$$

where $\Delta R(m,\tau_{n})$ represents the range difference from the antenna phase center to the target and scene origin. *K* and *c* represent the number of frequency samples in the phase history data of each pulse and the speed of light, respectively.

For the SAR frame generation with the range profile in Equation (7), interpolation is required to calculate the differential range accurately for every pixel from the discrete values of the range profile. This process can be implemented by performing linear interpolation on the inverse fast Fourier transform (IFFT) output of the zero-padded phase history data.

The final pixel response at location *r* is calculated by superposing all the interpolated values of the range profile, $s_{int}\left(r,\tau_{n}\right)$, with pulses within the synthetic aperture for the formation of the video frame. The pixel value at location *r* formed using *N* pulses starting from the *i*th pulse is given as follows.

(8) $$I\left(r,i,N\right)=\sum_{n=i}^{i+N-1}s_{int}\left(r,\tau_{n}\right).$$

For BP-VISAR, we propose constructing a sub-aperture consisting of adjacent pulses with a size less than that of the full-aperture. If the number of pulses in a sub-aperture is $N_{s}$ and *M* sub-apertures construct a full-aperture, the BP-VISAR frame for the sub-aperture starting from the *i*th pulse is defined as follows.

(9) $$I_{s}\left(r,i\right)=I\left(r,i,N_{s}\right).$$

Then, in the post-processing stage, the BP-VISAR frame for the full-aperture frame constructed by *M* sub-apertures starting from the *i*th pulse is defined as follows.

(10) $$I\left(r,i\right)=\sum_{m=0}^{M-1}I_{s}\left(r,i+m\cdot N_{s}\right).$$

By selecting the starting pulse positions for the full-synthetic-apertures for BP-VISAR frames, the overlapped or non-overlapped mode can be controlled. The offset between two starting pulse positions for the successive full-synthetic-apertures is $MN_{s}$ in a non-overlapped mode. On the other hand, the offset is less than $MN_{s}$ in an overlapped mode, depending on the overlap ratio.

### 3.2. Baseline Architectures of BP-VISAR

We present two baseline BP-VISAR architectures that adopt full- or sub-aperture processing on the computing platform equipped with a host processor and multiple GPU devices. We will use the notations in Table 1 for the description of the architectures.

Architecture F1 in Figure 3b is the baseline BP-VISAR for full-aperture processing. From the target cross-range resolution and the radar system parameters in Equation (6), the number of pulses $N_{p}$ required for the full-aperture can be obtained. This architecture partitions pulses into *M* disjoint groups of pulses that correspond to *M* sub-apertures. We allow the overlap of the sub-apertures between adjacent video SAR frames with the ratio of k/M. Range compression is performed on a pulse-by-pulse basis, and the range profiles for the pulses are stored in the sub-aperture range profile buffer (SRPB). After the range profiles are processed and stored for all aperture intervals, the image formation by the backprojection loop is initiated.

Architecture S1 in Figure 3c is the baseline BP-VISAR for sub-aperture processing. For each sub-aperture, the same BP-VISAR processing stages employed for the full-aperture processing are repeated such that low resolution BP-VISAR frames are produced for each sub-aperture. These are stored in the sub-aperture frame buffer (SFB) and superposed at a post-processing stage to generate a final BP-VISAR frame of target resolution.

In the non-overlapped mode in Figure 2a, there is no common pulse data shared between adjacent BP-VISAR frames both in Arch. F1 and Arch. S1. In this case, the generated frame rate may be insufficient for target tracking or moving target analysis unless the radar center frequency is shifted up into a higher band. In the overlapped mode in Figure 2b, range profiles in the SRPB used for image formation of the current frame can be reused for the image formation of the following frames. Moreover, the SFB used for the low-resolution BP-VISAR frame formation can be reused to generate the following frames.

To find the hotspots of computation in the BP-VISAR baseline architectures, we analyzed the computational complexities of the processing stages in Figure 3a for Arch. F1 and S1. Table 2 and Table 3 show the profiling results of the computational complexity of the BP-VISAR baseline architectures for one video SAR frame generation in the non-overlapped mode. We see that image formation takes most of the frame generation cycles and is the hotspot to accelerate.

The I/O stage performs the phase history data reception from the receiver. In our profiling environment, we used the Gotcha dataset for experiments to explore the BP-VISAR architecture [31]. The source bandwidth of the full-aperture processing BP-VISAR baseline architecture can be calculated from the receiver parameters as in the first row in Table 4. If the BP-VISAR processing system is fast enough, the source bandwidth of the phase history data is the actual I/O bandwidth in the system. Otherwise, in the computation-limited BP-VISAR system, the I/O bandwidth for the BP-VISAR architecture is constrained by the achievable frame rate of the BP-VISAR processing system. The same applies to the baseline architecture with sub-aperture processing. The bandwidth for the output frame storing process depends on the pixel resolution and the frame rate. In a modern computing system, the memory bandwidth is high enough to handle the I/O bandwidth required for BP-VISAR, and thus the processing cycles for I/O occupy a small portion of the overall processing time.

The processing cycles of the range compression stage are relatively minor compared to that of the image formation. It consists of IFFT of the phase history data after zero padding, rearrangement of the range profile, and the multiplication of phase ramp data. It is subsequently repeated on a pulse by pulse basis.

The image formation stage is shown to take most of the processing cycles regardless of the aperture processing type and the output pixel resolution. The image formation consists of range calculation, interpolation of the range profile, and accumulation of the interpolated ranges over the pulses. These operations are repeated for every pixel position to generate a BP-VISAR frame.

### 3.3. Architectures for BP-VISAR with Full-Synthetic-Aperture Processing

The output of the radar receiver, i.e., the PH data, has *K* frequency samples per pulse. The image formation stage calculates the pixel responses using the range profile data taken from a group of pulses corresponding to the full or sub-synthetic-apertures. However, the range profile data per pulse is not large enough to achieve memory access efficiency. Thus, we designate the range profile data generated from a group of pulses constituting a sub-synthetic-aperture as a processing data unit and design the data transmission and processing structure for BP-VISAR accordingly. The number of pulses for the processing data unit can affect the overlap ratio of synthetic apertures for BP-VISAR and the memory transaction efficiency. In this study, we set the unit size $N_{s}$ as 117, the number of pulses in one PH data file obtained within one-degree aperture angle in the Gotcha dataset.

**Figure 4:**
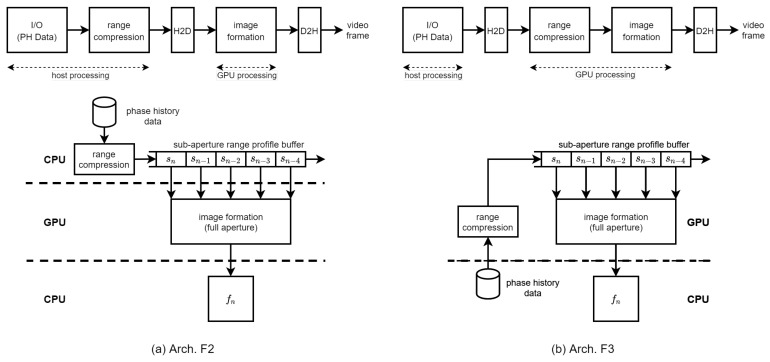
BP-VISAR: full-aperture processing architectures.

The group of phase history data $S(f_{k},\tau_{n})$ is range compressed either in the host CPU system in Arch. F2 or in the GPU device in Arch. F3 in Figure 4. In Arch. F2, the range profile data for each processing unit of pulses is queued into the host system’s sub-aperture range profile buffer. If the range profile data for *M* processing units are available for one image formation, they are transferred to the memory in the GPU device. Then, the image formation kernel is launched to calculate the pixel responses using the range profile data.

In Arch. F3, the range compression stage is performed in a GPU device kernel. Thus, the phase history data corresponding to the full-synthetic-aperture should be transferred to the GPU device for range compression. Because Arch. F3 locates the SRPB in the GPU device, it requires less host-to-device (H2D) bandwidth than that of Arch. F2 in the overlapped mode. The range compression is performed in a GPU kernel using cuFFT library functions. The generated range profile data unit is queued into the SRPB in the GPU device using the device-to-device (D2D) memory copy function. If the range profile data for *M* processing units are available in the SRPB, the image formation kernel is launched to calculate the pixel responses. Although the platform parameters, such as the positions of the platform and the radar system parameters, are transferred to the GPU device at the initial phase or periodically, their bandwidth is negligible compared to those of the PH data or range profile data. Generated SAR frames are transferred back to the host using the device-to-host (D2H) memory transfer CUDA functions.

In the CUDA implementation environment, there is a limit to the number of threads per block since all threads of a block are expected to reside on the same streaming multiprocessor (SM) and must share the limited memory resources of that SM. The total number of threads is equal to the number of threads per block times the number of blocks. In the CUDA implementation of Arch. F2 and F3, for the image formation, the maximum number of threads per SM is 1536 in the compute capability 8.6 of the Nvidia Ampere architecture [32]. The number of threads for image formation kernel execution is set to the number of pixels in the output video SAR frame to fully utilize the CUDA cores. This configuration can drive the occupancy of SMs up to 100%. Thus, to generate the video SAR frame of $N_{v}\times N_{v}$ pixel resolution, we constructed blocks with a 16×16 size and a grid consisting of $N_{v}/16\times N_{v}/16$ blocks. The range compression kernel consisted of CUDA functions performing zero paddings, IFFT, and scaling and shifting of range profile. The range compression operated on a per-pulse basis with $N_{IFFT}/1024$ one-dimensional blocks of 1024 threads.

For the host-to-device data transfer of the range profile data in the SRPB in Arch. F2 or the phase history data of one sub-aperture unit in Arch. F3, the global memory in the GPU device is used. The range profile data are managed on a sub-aperture unit basis in the SRPB to support both non-overlapped and overlapped modes in BP-VISAR to improve the frame rate. In addition, the SRPB buffer operates in a first-in-first-out (FIFO) mode to keep the recent sub-aperture units constructing a new full-aperture. The range profile data are accessed repeatedly by the kernels of the CUDA cores in the GPU to calculate the pixel response. Although the shared memory can be the candidate location of the SRPB for fast access by threads, its memory limit per block prevents threads from accessing other blocks. Hence, the SRPB is located in the global memory in our system.

For the device-to-host data transfer of output video frames in Arch. F2 and F3, the global memory in the GPU device is used. Each image formation kernel calculates its pixel responses by repeatedly accumulating the interpolated range profile data and writing the pixel responses to the output frames. The temporary variable of the pixel response repeatedly accessed by each kernel is stored in the register of the multiprocessor. The final output frame memory is located in the global memory section in the GPU device. Because the radar platform information, including the radar platform trajectory and radar system parameters, needs to be accessed by all the image formation kernel threads simultaneously, it is located in the shared memory for fast access.

For comparison, the bandwidth between the processing stages in Figure 3a is summarized in Table 4 for non-overlapped and overlapped modes. For fast data transfer between the host and the GPU device, we used the asynchronous data transfer method controlled by CUDA streams by allocating pinned memory, i.e., page-locked memory, in the host for the data transmitted to or received from the GPU device. All the transferred data for the BP-VISAR can be allocated as the page-locked memory because it can fit within the size of the RAM in the host; for example, the minimum page-locked memories required for 4096 × 4096 frame resolution are 153 MB and 135 MB for Arch. F2 and F3, respectively. Although the host-to-device bandwidth required for range profile data transfer in Arch. F2 is not affected by the overlap mode, it depends on the overlap ratio α in Arch. F3. If α equals zero, these architectures operate in a non-overlapped mode. For the given target frame rate, the device-to-host bandwidth for output frame transfer is the same for both Arch. F2 and F3 modes.

All the bandwidths required for the continuous operation of the BP-VISAR algorithm with the target frame rate can be calculated with actual data. For example, for a frame resolution of 4096×4096 and a target frame rate of 10, the required H2D and D2H bandwidths for Arch. F2 and Arch. F3 in the non-overlapped mode are 1.53 GB/s and 1.36 GB/s, respectively. Suppose we use the overlapped mode with an overlap ratio of 80%, the bandwidth for Arch. F3 is reduced to 1.35 GB/s, but the bandwidth for Arch. F2 is the same as that in the non-overlapped mode. The PCIe 4.0x16 is used for the H2D channel and provides a bandwidth of 31.5 GB/s. Thus, the data transfer channel between the host and the device can provide sufficient bandwidth for the BP-VISAR systems.

### 3.4. Architecture Exploration for BP-VISAR with Sub-Aperture Processing

The baseline architecture for BP-VISAR with sub-aperture processing performs all the computation and memory I/O in the host system. It is distinguished from the full-aperture processing by the size of the range profile data for image formation and the sum of sub-aperture images at the end of the processing.

**Figure 5:**
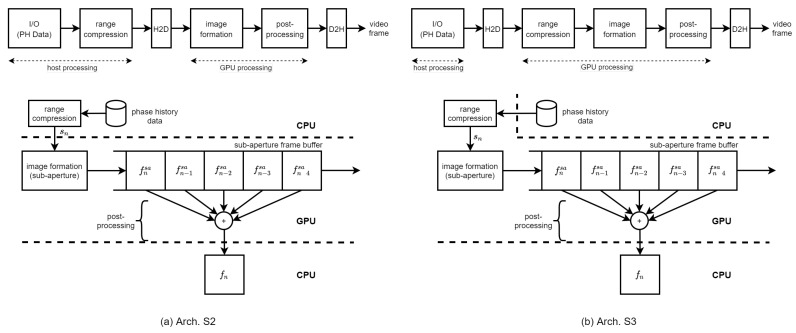
BP-VISAR: sub-aperture processing architectures.

We propose two BP-VISAR architectures with sub-aperture processing in Figure 5. The difference of Arch. S2 and S3 from Arch. F2 and F3 with full-aperture processing is the size of the input to the IF stage. The image formation stages in Arch. S2 and S3 generate multiple frames of low resolution for every sub-aperture processing unit. These frames of low resolution are queued into the sub-aperture frame buffer to generate higher resolution frames in the post-processing stage. Finally, the post-processing stage sums all the frames in the SFB into the final frame of high resolution, which is equivalent to the result by the full-synthetic-aperture processing. Because the computational complexity of the image summation in the post-processing stage is highly relieved compared with that of the image formation in Table 3, the post-processing is integrated into the image formation kernel.

The difference between Arch. S2 and S3 is the location for the range compression. The group of phase history data is range compressed either in the host system in Arch. S2 or in the GPU device in Arch. S3. In both architectures, the range profile data of a processing unit is temporarily stored in the global memory in the GPU device before the image formation stage without the SRPB.

For fast data transfer between the host and the GPU device, we used the asynchronous data transfer method controlled by CUDA streams by allocating pinned memory as in the architectures for full-aperture processing. All the transferred data for the BP-VISAR is within the size of the RAM and can be allocated as the page-locked memory; for example, the minimum page-locked memories required for a 4096 × 4096 frame resolution are 138 MB and 135 MB for Arch. S2 and S3, respectively.

All the construction of grids and blocks of threads for the range compression kernel and the image formation kernel are the same as in full-aperture processing. For the H2D data transfer of the range profile data in Arch. S2 and the phase history data of one sub-aperture unit in Arch. S3, the global memory in the GPU device is used. The SFB buffer operates in FIFO mode to employ the recent sub-aperture frames for constructing the next full-aperture. For the D2H data transfer of output video frames in Arch. S2 and S3, the global memory in the GPU device is used. Each image formation kernel generates the final frame of the target resolution by summing all the low resolution frames in the SFB while calculating its pixel response by repeatedly accumulating the interpolated range profile data and writing the pixel response to the output frame. Because the radar platform information, including the radar platform trajectory and radar system parameters, is accessed by all the image formation kernel threads simultaneously, it is located in the shared memory for fast access.

The bandwidth between the processing stages in Figure 3c is summarized in Table 5 for both non-overlapped and overlapped modes. For a given frame rate, the host-to-device bandwidth is proportional to the frame rate and is dependent on the overlap ratio. The device-to-host bandwidth for output frame transfer is the same for all the architectures, F2, F3, S2, and S3.

The bandwidths required for the continuous operation of the BP-VISAR can be calculated from the frame resolution and frame rate. For a frame resolution of 4096×4096 and a target frame rate of 10, the required bandwidths for Arch. S2 and Arch. S3 in the non-overlapped mode are 1.53 GB/s and 1.36 GB/s, respectively. If we use the overlapped mode with an overlap ratio of 80%, the bandwidth for Arch. S2 and Arch. S3 are reduced to 1.38 GB/s and 1.35 GB/s, respectively. Thus, the data transfer channel between the host and the device provides sufficient bandwidth for the BP-VISAR systems.

### 3.5. Multi-Stream Based BPA Acceleration

A GPU device has an execution engine, copy engines, a memory controller, and high-speed memory. The execution engine consists of many parallel processors called a multiprocessor in Nvidia terms. The copy engines are connected to the PCIe bus and transmit data between the host and GPU using direct memory access (DMA) controllers. The GPU device used for our study has one execution and six copy engines. Because these engines work independently, their operation can be overlapped in different streams.

A stream in CUDA is a sequence of operations that execute on the device in the order in which the host issues them. All device operations, including kernels and data transfers in CUDA, run in a stream. Therefore, the BP-VISAR algorithm is executed in a stream in the order of H2D data transfer, range compression, image formation, and D2H data transfer. The execution stages of BP-VISAR can be categorized into two groups according to the hardware used in the device. The first is the computation group consisting of the range compression, image formation, and post-processing executed by the execution engine. The second is the data transfer group consisting of the H2D and D2H data transfers, which invokes the copy engines responsible for controlling DMAs between the host and the device irrespective of the CUDA kernel engine.

All the BP-VISAR architectures with full or sub-aperture processing are designed in a single stream as in the previous sections. In a single stream execution of the BP-VISAR pipeline, because the operations of copy or kernel engines are blocked by the others, the execution engine and copy engines are not fully utilized. This is because the execution and data transfer operations are running in the order shown in Figure 4 and Figure 5. For example, the execution engine may be idle if the range profile data is not transferred from the host in Arch. F2 and Arch S2. The execution engine cannot run for the next frame generation until the current frame is generated and transferred to the host. However, it is likely that the copy engines stay mostly in an idle mode because the BP-VISAR pipeline is computation-limited.

**Figure 6:**
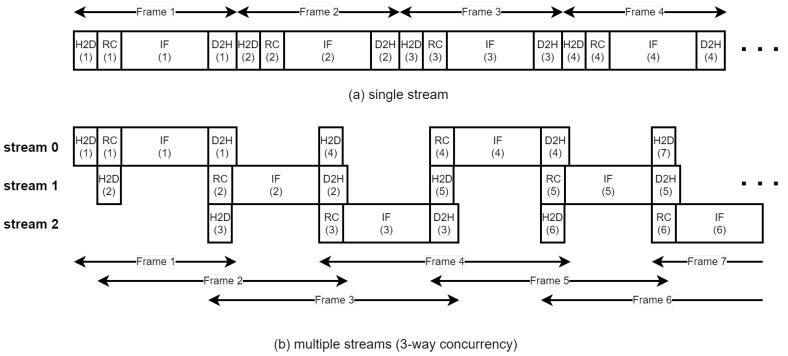
Multiple stream processing of BP-VISAR.

Therefore, if the kernel execution and the data transfer in different streams overlap, the overall throughput can be improved over the single-stream architecture. To improve the performance of BP-VISAR, we propose a multi-stream BP-VISAR architecture with N-way concurrency as in Figure 6b, which overlaps kernel execution and data transfers. We selected Arch. S3 as the reference single stream architecture due to its prominent performance, which is explained in the later section. The multi-stream processing architecture of BP-VISAR, Arch. S3-N, is designed by enabling N-way concurrency for generating video frames. The execution process of the BP-VISAR is not bandwidth-limited but computation-limited from the profiling results in Table 2 and Table 3 or the performance measurement of Arch. S3, which is presented in the experimental section. Kernels are queued into the compute queue and executed in the order of their launching times. While executing the image formation kernel in the GPU, the execution engine allocates all the available CUDA cores to the kernel threads because the number of threads for the kernel is larger than the number of CUDA cores in the GPU device. Although simultaneous execution of the kernels may exist in different streams at the starting or ending phase of the kernels, its contribution to performance enhancement is minimal as the rate of CUDA cores transitioning from idle to the active state by simultaneous kernel execution is not high.

However, the performance can be improved by overlapping the kernel execution in a stream with H2D or D2H data transfer in different streams. In Figure 6b, although only one kernel from the streams is executed at any instance, its execution can be overlapped with H2D or D2H data transfer if there is any data for transfer in the other streams. Throughout the concurrent execution of the kernel and data transfers, the overall execution time can be reduced. The number of streams that maximizes the operating speed of BP-VISAR is explored by varying the number of streams, and the results are presented in the experimental section. In Arch. S3-N, the stream *k* generates video frames of the same residue *k* after modulo-N operation of its frame number. The host memory involved in data transfer is allocated as the pinned memory to enable the asynchronous data transfer. We use dedicated H2D and D2H memory space for each stream to prevent data corruption due to memory access by other streams.

### 3.6. Multi-Stream Based BPA on Multiple GPUs

**Figure 7:**
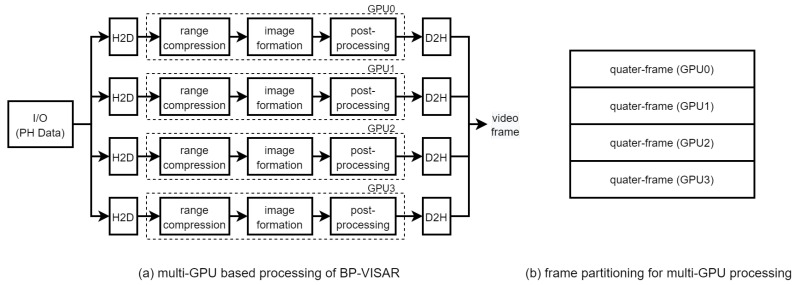
Multi-GPU processing of Arch. S3: stream processing.

The proposed architectures can be extended to be scalable to the available number of GPU devices. For the continuous operation of BP-VISAR for streaming phase history data input, we propose a multi-GPU based BP-VISAR architecture as in Figure 7a, which shows the case of four GPU devices. In each GPU, BP-VISAR operates in Arch. S3-N, and receives the streaming phase history data as input and generates quarter video frames as in Figure 7b. Because all the GPUs take the same streaming input and perform the range compression on the same data, this computation can be redundant. However, this architecture can remove the bandwidth overhead for the distribution of the range profile data from a GPU performing the range compression to the others. Moreover, this redundant range compression in each GPU does not diminish the benefit of distributing the image formation load to multiple GPUs because the computation time of the range compression is negligible compared to that of the image formation. In the proposed multi-GPU architecture, the frame merge operation at the end of each quarter-frame generation can be removed just by adjusting the target addresses for D2H transfer of quarter-frames with the stride of the quarter-frame size.

## 4. Experimental Results

In this section, we present the performance analysis and the experimental results for the explored BP-VISAR architectures.

### 4.1. Experimental Environments

The proposed architectures were implemented on a system equipped with Intel Xeon CPU with 128-GB main memory and four Nvidia RTX 3090 GPUs. The GPU has 82 streaming multiprocessors and 128 CUDA cores per SM. It has six copy engines and 24-GB global memory. By taking the I/O, range compression, and image formation modules from the open-source [33], we implemented the baseline architectures of Arch. F1 and S1 in C/C++. The overall performances of the baseline architectures were used as references for performance comparison. We implemented the full-aperture and the sub-aperture processing architectures in C/C++ for multi-thread parallelization in GPU devices. Starting from the baseline architectures, we migrated the range compression and the image formation from the C/C++ codes to CUDA C codes in Microsoft Visual Studio 2019 and CUDA Toolkit 11.3. The RC stage consisted of the H2D data transfer and the RC kernel. The IF stage consisted of the IF kernel and the D2H data transfer. All variables for RC and IF kernels are of float data type.

To evaluate the performance of the BP-VISAR architectures, we used the AFRL Gotcha circular SAR dataset, which consisted of eight complete circular passes, with each pass at a different elevation angle. The dataset consisted of SAR phase history data collected at X-band with a 640 MHz bandwidth [31]. The phase history data collected for one degree of azimuth was stored in a file in the dataset.

We set $N_{s}$ to 117, the number of pulses corresponding to one degree of azimuth in the dataset. We set $N_{p}$ to 585, which was the five multiples of $N_{s}$. This configuration of synthetic aperture length resulted in the cross-range resolution of 0.26 m for video SAR frames. Different numbers of $N_{s}$ and $N_{p}$ were configured for different cross-range resolutions or computational loads considering the requirements for target BP-VISAR applications.

The average elapsed times for generating one BP-VISAR frame were measured for the architectures explored in non-overlapped and overlapped modes. For efficient target tracking, it is necessary to increase the overlap ratio more or raise the center frequency. The choice of the overlap ratio does not affect the imaging quality. However, if the overlap ratio is too close to 100%, the newly generated video SAR frame may not have sufficient changed information for target tracking but waste the computational resources. The overlap ratio of 80% was used for the overlapped mode. The target frame rate needed to be set according to the requirements of the BP-VISAR system applications under certain limitations, such as the cross-range resolution and the computational resources. Therefore, we evaluated the performance of the BP-VISAR architectures in the best-effort mode in which the architectures run as fast as possible. This evaluation method measured the performance improvement by the architectures that were explored relative to the reference BP-VISAR architectures in a given computing platform.

To investigate the changes in the visual quality of video SAR frames during the acceleration process, we used the PSNR to compare the quality of video SAR frames generated in each architecture with those in the baseline architectures.

### 4.2. Performance Analysis of BP-VISAR Architectures with Full-Aperture Processing

**Figure 8:**
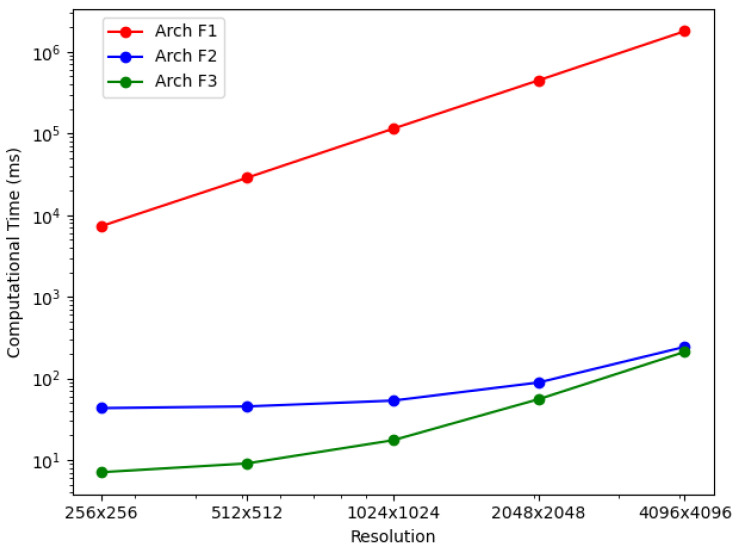
BP-VISAR frame generation time with full-aperture processing in overlapped mode (α=0.8).

For the BP-VISAR architectures with full-aperture processing, the elapsed times for generating video SAR frames were measured for non-overlapped and overlapped modes. The elapsed times were averaged along the circular flight path of the Gotcha dataset and are summarized in Table 6 and Figure 8. The column *speedup* in Table 6 and Table 7 represents the ratio of the processing time of the baseline architecture to that of the proposed architecture.

In Arch. F1, the average elapsed time increases dramatically with the pixel resolution of BP-VISAR frames, which is a typical phenomenon in conventional backprojection algorithms [2,6,34,35]. Because all the frames of different pixel resolutions use the same range profile data for a given configuration of synthetic aperture processing and most of the computational complexity is from the IF stage as shown in Table 2, the computational loads are approximately proportional to the $N_{v}^{2}$.

In Arch. F2 and F3 with non-overlapped and overlapped modes, we measured elapsed times for each processing stage to analyze how the new architectures affect each stage’s processing time and overall time. It can be seen that Arch. F2 and F3 show significant improvement in overall processing times over Arch. F1. As can be inferred in the profile data in Table 2, most of the acceleration is from the parallelization of the IF stage based on the nature of the independent pixel response calculation. The accelerated processing time in the IF stage is still proportional to the number of pixels in the frame regardless of the overlap mode. Although the RC stage is also parallelized in Arch. F3, its effect on the overall processing time decreases, and the IF stage contributes most of the overall processing time for higher frame resolution.

In Arch. F3, compared with Arch. F2, there is further acceleration in the RC stage by the parallelization in the GPU device regardless of the overlap mode. The range compression times for Arch. F3 are reduced to about 7% and 8% of those of Arch. F2 in non-overlapped and overlapped modes, respectively. This time reduction is from using CUDA threads to parallelize the IFFT, scaling, and shift operations. The amount of time reduction in range compression is the same for all frame resolutions because range compression depends only on the dimensions of the phase history data and the number of the range profile bins.

The RC times for Arch. F2 and F3 in the overlapped mode are reduced from those in the non-overlapped mode by a factor of 4.95 and 6.34, respectively. Five sub-apertures construct one full-aperture for target cross-range resolution. For the overlap ratio of 80% in Table 6 (b), only one processing data unit is handled by the RC stage compared with five units in the non-overlapped mode. Parallelization of the RC stage in Arch. F3 further accelerates the overall performance of Arch. F2 by a factor of 1.7–6.0 and 1.2–6.1 for the non-overlapped and the overlapped modes, respectively.

The I/O time is independent of the architectures but depends on the overlap mode. In the same overlap mode, the I/O times for all architectures are the same because there is no change in loading the phase history data from the storage. However, the I/O time for the overlapped mode is reduced by the factor *M* from the non-overlapped mode. Only the newly required phase history data for the current synthetic aperture should be loaded in the overlapped mode.

**Figure 9:**
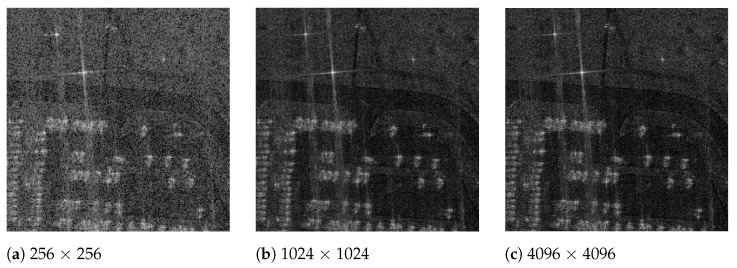
BP-VISAR frames of different frame resolutions (full-aperture processing in overlapped mode, frame 7, α=0.8).

Figure 9 shows the generated BP-VISAR frames of different frame resolutions for the Gotcha dataset. The visual quality is enhanced with a higher frame resolution. Although the proposed architectures accelerate the processing significantly, there is no severe degradation in the PSNR of the generated BP-VISAR frames compared with the baseline architecture.

### 4.3. Performance Analysis of BP-VISAR Architectures with Sub-Aperture Processing

**Figure 10:**
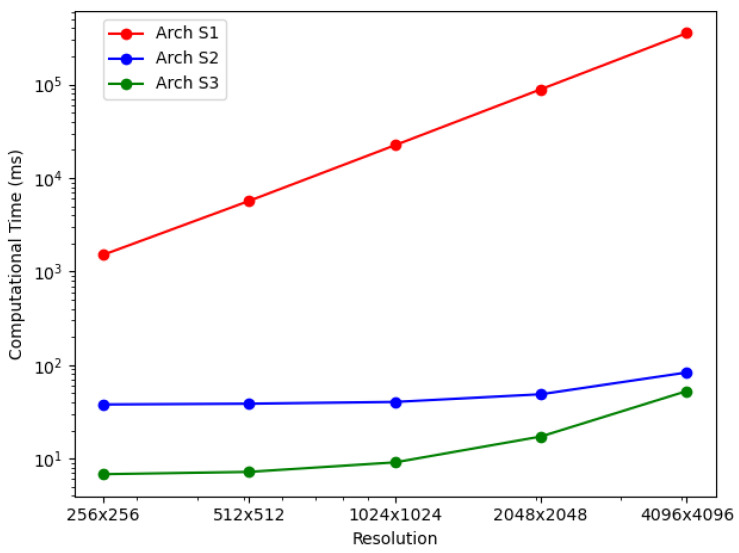
BP-VISAR frame generation time with sub-aperture processing in overlapped mode (α=0.8).

For the BP-VISAR architectures with sub-aperture processing, the elapsed times for generating video SAR frames were measured for non-overlapped and overlapped modes. The average elapsed times are summarized in Table 7 and Figure 10.

In Arch. S1, regardless of the overlap mode, the average elapsed time also increases with the BP-VISAR frame resolution, and most of the computational complexity is from the IF stage as in Arch. F1. Arch. S2 and S3 show significant improvements in overall processing times over Arch. S1. As can be inferred in the profile data in Table 3, most of the acceleration is also from the parallelization of the IF stage. Once the IF stage is parallelized, it does not use most of the overall processing time as in Arch. S1.

In non-overlapped mode, for one frame generation, the sub-aperture processing performs the IF stage *M* times with the range profile data for one processing data unit. In contrast, the full-aperture processing performs the IF stage once with the range profile data for *M* processing data units. Arch. F1 and S1 perform all the processing in the host system while Arch. F2 and S2 perform the IF stage in the GPU device and Arch. F3 and S3 perform the RC and the IF stages in the GPU device. Despite the architectural differences in full- and sub-aperture processing, such as the SRPB and SFB, the overall processing times of Arch. S1 to S3 are not significantly different from those of Arch. F1 to F3.

In the overlapped mode, for one frame generation, the architectures of sub-aperture processing perform the IF stage once with the range profile data for one processing data unit. In contrast, the architectures of full-aperture processing do the same processing in the non-overlapped mode except for the reduced execution of the RC stage. Because the IF stage accounts for the majority of the total computation in the generation of high-resolution frames, the reduced number of executions in the IF stage can significantly impact the overall processing time. For the overlap ratio of 0.8 in Table 6 (b) and Table 7 (b), the overall processing times of Arch. S1 to S3 are improved by ratios of 2.9–4.5 compared with those of Arch. F1 to F3. In Arch. S3, compared with Arch. S2, there is further acceleration in the RC stage by the parallelization in the GPU device regardless of the overlap mode. The I/O time is independent of the architectures but depends on the overlap mode.

**Figure 11:**
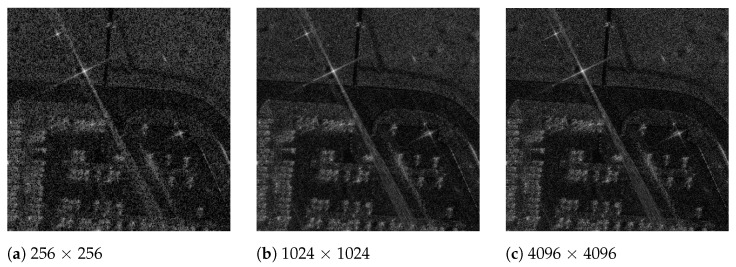
BP-VISAR frames of different frame resolutions (sub-aperture processing in overlapped mode, frame 29, α=0.8).

Figure 11 shows the generated BP-VISAR frames of different frame resolutions for the Gotcha dataset. The visual quality is enhanced with a higher frame resolution. Although the proposed architectures accelerate the processing significantly, there is no severe degradation in the PSNR of the generated BP-VISAR frames compared with the baseline architecture.

### 4.4. Performance Analysis of BP-VISAR Architectures with Multi-Stream Based Acceleration

To evaluate the performance of the multi-stream based BP-VISAR architectures, we implemented N-way concurrent Arch. S3, i.e., Arch. S3-N by grouping the output frames and allocating the tasks of generating the groups of the frames to the concurrent streams of the BP-VISAR pipeline. We measured the overall processing times and visually profiled the internal status of the GPU device for Arch. S3 and Arch. S3-N.

**Figure 12:**
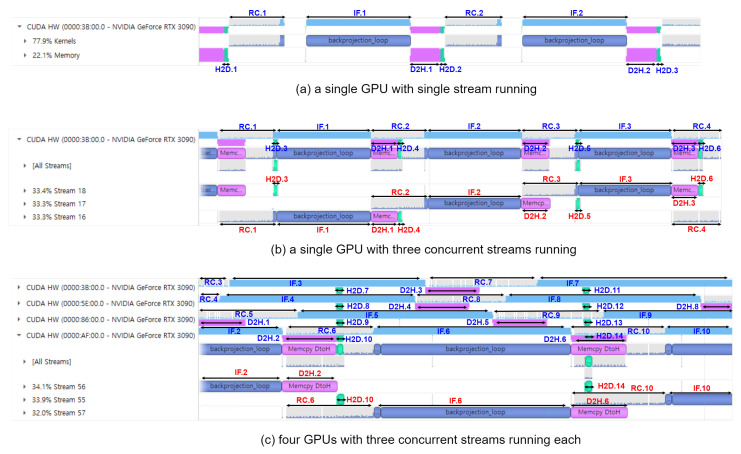
Visual profiling results of Arch. S3 and its multi-stream and multi-GPU extensions. Suffix *n* of H2D.*n*, RC.*n*, IF.*n*, D2H.*n* represents the BP-VISAR frame number. Target frame resolutions are 2048 × 2048 for (**a**,**b**) and 4096 × 4096 for (**c**), respectively.

Arch. S3 is executed as one stream in the GPU device as in Figure 12a where kernels and memory data transfer are executed in the issue order by the host. Therefore, when the execution engine runs RC or IF kernels for the *n*th frame, the copy engine is idle, and vice versa.

Arch. S3-3 is executed as three streams in the GPU device as in Figure 12b. The kernels and memory data transfers in each stream are still executed in the issue order by the host. However, there is simultaneous execution of a kernel and memory data transfer from different streams. For example, the RC kernel for frame 2 (RC.2) operates simultaneously with the memory data transfer for frame 1 (D2H.1) and frame 4 (H2D.4). Memory data transfer for frame 5 (H2D.5) operates simultaneously with the RC and the IF kernels for frame 3 (RC.3 and IF.3), etc. These concurrent executions of the execution engine and the copy engine can further accelerate the overall processing time by increasing the resource utilization of the GPU device. As a result, there is performance improvement in Arch. S3-3 by 6–31 % and 12–38 % in non-overlapped and overlapped modes compared to Arch. S3, respectively.

For the multi-stream execution of BP-VISAR, a number of streams saturate the performance due to the finite resources of the execution and the copy engines in the GPU device. The number is dependent on the structure of the BP-VISAR pipeline in a stream and the GPU resources. Table 8 shows the average processing time for one BP-VISAR frame generation with the number of streams in Arch S3-N. In Arch. S3-N, three or four streams of the BP-VISAR pipeline are optimal for speed. In this study, we used three streams for Arch. S3-N for the performance comparison with others.

### 4.5. Performance Analysis of the Multi-GPU Based Streaming BP-VISAR Architecture

To evaluate the performance of the multi-GPU based BP-VISAR architecture, we implemented the streaming architecture of four GPUs in Figure 7a. All the GPUs launched three streams of the BP-VIPSAR pipeline in Arch. S3-3 simultaneously. Only the size and the dimensions of the output frame generated were different from the single GPU based architecture Arch. S3-3 as in Figure 7b.

**Figure 13:**
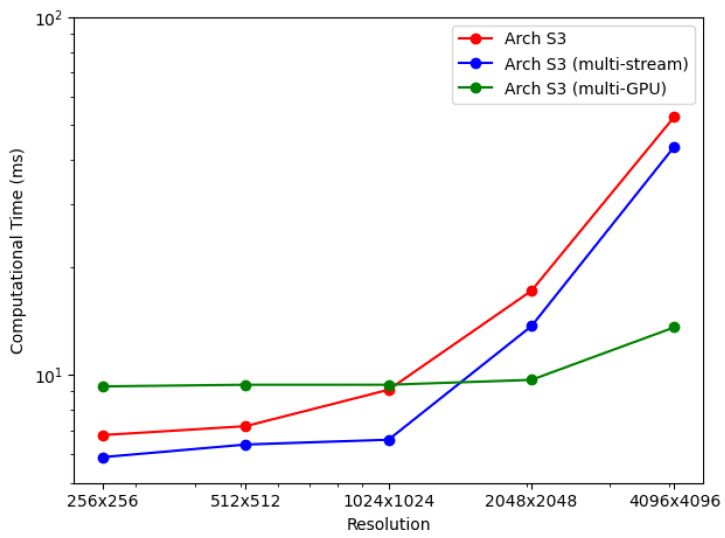
Performance comparison: multiple stream and multi-GPU processing.

Table 7 and Figure 13 show the processing times of multi-GPU based streaming architectures for non-overlapped and overlapped modes. For lower resolution BP-VISAR frame processing, the processing times per frame are around 50 ms for lower resolutions of video SAR frames. Compared with the processing times in Arch. S3-3 with three streams running on a single GPU, there is no advantage to using the multi-GPU based architecture for resolutions less than 1024 × 1024, but there is an increased cost for quad-GPU. However, the processing is accelerated by 3.2 times both for non-overlapped and overlapped modes in 4096 × 4096 resolution. From the results, for lower resolution video SAR frame processing, the overhead for frequent control of the multi-GPU data transfer and kernel execution is too large to take advantage of multi-GPU based parallel processing. However, for higher resolution video SAR frame processing, the frequency for controlling the multi-GPU data transfer and kernel execution decreases due to the longer backprojection and data transfer times.

Figure 12c shows the visual profiling timeline for executing the quad-GPU streaming BP-VISAR architecture. There are time lags between the starting times of the pipelines of the GPUs. However, except for the starting time lag, there is no dependency between the operation of the pipelines in the GPUs because the pipelines are not bandwidth limited but computation limited. Although the H2D and D2H data transfer in a GPU may conflict with the data transfer in the other GPUs, the bandwidth between the host and the GPUs is sufficient for fast data transfer. Thus, the latency caused by the bus contention is negligible.

### 4.6. Discussion

Conventional studies on GPU-based BPA acceleration have mainly presented the imaging time and speed-up ratio after acceleration as performance measures [20,23], but these studies did not perform comparative studies with the previous literature of different GPU computing and SAR imaging environments.

For objective comparison of acceleration performance, excluding the effect of different GPUs and the SAR imaging parameters, we define the normalized imaging time as follows.

(11) $$t_{norm}=t_{i}\cdot \frac{n_{core}\cdot f_{GPU}}{n_{pixel}\cdot N_{p}},$$

where $t_{i}$, $n_{core}$, $f_{GPU}$, $n_{pixel}$ represent the imaging time of one SAR image or frame, the number of CUDA cores, the clock frequency of the GPU, and the number of imaging pixels, respectively.

Table 9 shows the normalized imaging times for BPA acceleration architectures. We set the imaging time of Arch. F3 of the non-overlapped mode as the reference for comparison. The GPU accelerated BPA architectures in references [20,23] are 1.49 and 1.13 times slower than the proposed Arch. F3 with non-overlapped aperture processing. For the proposed architectures, increasing the overlap ratio helps reduce the normalized time, especially for the sub-aperture processing architecture of Arch. S3. For Arch. S3, multi-stream processing within a single GPU helps utilize the GPU resources efficiently, and the processing speed is scalable to the number of GPUs.

## 5. Conclusions

This paper proposed architectures for backprojection based video SAR for a system equipped with multiple GPUs. We analyzed the video SAR frame rate for non-overlapped and overlapped aperture modes. Depending on the choice of processing aperture length and the parallel structure on GPU devices, six architectures of single-stream BP-VISAR pipeline were proposed and their performances evaluated in both non-overlapped and overlapped modes. The GPU parallelized architecture with sub-aperture processing were found to be the best architecture as a single-stream pipeline and its architecture was further extended for multiple streams and multiple GPUs to utilize the idle GPU resources further. The experimental results showed that the proposed BP-VISAR architectures with sub-aperture processing were scalable with GPU devices for high pixel resolution.

The proposed BP-VISAR architectures can be used to generate video SAR frames of target cross-range resolution in real-time from the streaming phase history data of the radar sensor. The accelerated video SAR systems can be loaded on airborne or drone platforms for collaborative day-and-night surveillance on the video SAR sensor networks.

## Figures and Tables

**Table 1:** Notations.

Symbol	Description
$N_{p}$	the number of pulses constructing a full-synthetic-aperture
$N_{s}$	the number of pulses constructing a sub-synthetic-aperture
$N_{IFFT}$	the number of inputs for IFFT in the range compression stage
$N_{v}$	the number of pixels in rows or columns in video SAR frame of square size
*K*	the number of frequency samples in the phase history data of each pulse
*M*	the number of sub-synthetic-apertures constructing a full-synthetic-aperture
$f_{PRF}$	the pulse repetition frequency in a radar platform
α	the overlap ratio of the synthetic apertures for adjacent video SAR frames (=k/M)
γ	the frame rate

**Table 2:** The computational complexity of BP-VISAR baseline architecture F1.

Processing Stage	256 × 256		1024 × 1024		4096 × 4096	
	Time (ms)	%Time	Time (ms)	%Time	Time (ms)	%Time
I/O	17	0.218	19	0.017	19	0.001
range compression	291	3.737	281	0.245	278	0.015
image formation	7478	96.044	114,254	99.738	1,797,197	99.983

**Table 3:** The computational complexity of BP-VISAR baseline architecture S1.

Processing Stage	256 × 256		1024 × 1024		4096 × 4096	
	Time (ms)	%Time	Time (ms)	%Time	Time (ms)	%Time
I/O	21	0.271	21	0.019	22	0.001
range compression	263	3.389	292	0.258	301	0.017
image formation	7476	96.328	112,748	99.713	1,782,637	99.970
post-processing	1	0.013	12	0.011	206	0.012

**Table 4:** Bandwidth and buffer sizes in the BP-VISAR architectures with full-aperture processing.

Processing Stage/Buffer		Non-Overlapped	Overlapped
I/O (source)	bandwidth	$8Kf_{PRF}$	$8Kf_{PRF}$
I/O (feed)		$8KN_{p}\gamma$	$8KN_{p}\left(1-\alpha \right)\gamma$
range compression		$8N_{IFFT}N_{p}\gamma$	$8N_{IFFT}N_{p}\left(1-\alpha \right)\gamma$
image formation		$8N_{v}^{2}\gamma$	$8N_{v}^{2}\gamma$
SRPB(out)		$8N_{IFFT}N_{p}\gamma$	$8N_{IFFT}N_{p}\gamma$
SRPB	buffer size	$8N_{IFFT}N_{p}$	$8N_{IFFT}N_{p}$

**Table 5:** Bandwidth in the BP-VISAR architectures with sub-aperture processing.

Processing Stage/Buffer		Non-Overlapped	Overlapped
I/O (source)	bandwidth	$8Kf_{PRF}$	$8Kf_{PRF}$
I/O (feed)		$8KN_{p}\gamma$	$8KN_{p}\left(1-\alpha \right)\gamma$
range compression		$8N_{IFFT}N_{p}\gamma$	$8N_{IFFT}N_{p}\left(1-\alpha \right)\gamma$
image formation		$8N_{v}^{2}M\gamma$	$8N_{v}^{2}M\left(1-\alpha \right)\gamma$
post-processing		$8N_{v}^{2}\gamma$	$8N_{v}^{2}\gamma$
SFB	buffer size	$8N_{v}^{2}M$	$8N_{v}^{2}M$

**Table 6:** Processing times in BP-VISAR architectures with full-aperture processing. (**a**) Non-overlapped mode, α=0, unit: ms, dB. (**b**) Overlapped mode, α=0.8, unit: ms, dB.

(**a**)							
	**Resolution**	**I/O**	**RC**	**IF**	**SUM**	**Speedup**	**PSNR**
Arch. F1	256 × 256				7785.0	1	n/a
	512 × 512				29,205.0	1	n/a
	1024 × 1024				114,554.1	1	n/a
	2048 × 2048				451,459.5	1	n/a
	4096 × 4096				1,797,493.5	1	n/a
Arch. F2	256 × 256	16.6	186.4	2.9	205.8	38	42.2
	512 × 512	16.4	184.8	5.1	206.3	142	45.3
	1024 × 1024	16.5	183.2	13.4	213.1	538	45.6
	2048 × 2048	16.6	183.6	48.8	249.0	1814	45.8
	4096 × 4096	16.2	183.0	203.1	402.3	4468	45.8
Arch. F3	256 × 256	16.4	16.7	1.2	34.4	227	42.2
	512 × 512	16.5	16.6	3.6	36.6	797	45.3
	1024 × 1024	16.8	16.3	11.8	44.9	2550	45.6
	2048 × 2048	16.0	16.6	47.6	80.2	5628	45.8
	4096 × 4096	16.6	16.5	202.3	235.5	7634	45.8
(**b**)							
	**Resolution**	**I/O**	**RC**	**IF**	**SUM**	**Speedup**	**PSNR**
Arch. F1	256 × 256				7355.1	1	n/a
	512 × 512				28,805.3	1	n/a
	1024 × 1024				114,631.5	1	n/a
	2048 × 2048				450,153.9	1	n/a
	4096 × 4096				1,794,174.3	1	n/a
Arch. F2	256 × 256	3.3	37.2	2.8	43.3	170	42.2
	512 × 512	3.2	37.1	5.2	45.4	635	45.3
	1024 × 1024	3.1	37.0	13.4	53.5	2142	45.6
	2048 × 2048	3.3	37.3	48.7	89.3	5042	45.8
	4096 × 4096	3.3	37.6	202.7	243.7	7363	45.8
Arch. F3	256 × 256	3.3	2.7	1.1	7.1	1037	42.2
	512 × 512	3.1	2.4	3.5	9.1	3176	45.3
	1024 × 1024	3.2	2.4	11.9	17.5	6550	45.6
	2048 × 2048	3.1	2.6	50.1	55.8	8066	45.8
	4096 × 4096	3.2	2.9	205.4	211.5	8483	45.8

**Table 7:** Processing times in BP-VISAR architectures with sub-aperture processing. (**a**) Non-overlapped mode, α=0, unit: ms, dB. (**b**) Overlapped mode, α=0.8, unit: ms, dB.

(**a**)							
	**Resolution**	**I/O**	**RC**	**IF**	**SUM**	**Speedup**	**PSNR**
Arch. S1	256 × 256				7760.8	1	n/a
	512 × 512				28,978.3	1	n/a
	1024 × 1024				113,072.5	1	n/a
	2048 × 2048				448,055.0	1	n/a
	4096 × 4096				1,783,164.8	1	n/a
Arch. S2	256 × 256	16.3	167.1	3.3	186.6	42	42.2
	512 × 512	16.5	166.3	5.5	188.2	154	45.3
	1024 × 1024	16.3	166.4	13.6	196.3	576	45.6
	2048 × 2048	16.5	164.9	46.8	228.2	1963	45.8
	4096 × 4096	16.4	164.8	182.8	363.9	4901	45.8
Arch. S3	256 × 256	16.3	15.5	1.2	33.1	235	42.2
	512 × 512	16.3	15.9	2.7	35.0	829	45.3
	1024 × 1024	16.8	15.4	10.9	43.1	2626	45.6
	2048 × 2048	16.4	15.6	43.7	75.7	5919	45.8
	4096 × 4096	16.8	15.4	195.1	227.4	7843	45.8
Arch. S3 (Multi-Stream)	256 × 256	17.4	13.6		31.0	250	42.2
	512 × 512	17.9	14.3		32.2	899	45.3
	1024 × 1024	17.6	15.3		32.9	3435	45.6
	2048 × 2048	17.4	48.8		66.2	6773	45.8
	4096 × 4096	17.3	196.9		214.2	8325	45.8
Arch. S3 (Multi-GPU)	256 × 256	16.7	32.5		49.2	158	42.2
	512 × 512	16.5	33.2		49.7	583	45.3
	1024 × 1024	17.2	34.5		51.7	2189	45.6
	2048 × 2048	17.8	36.2		54.0	8300	45.8
	4096 × 4096	16.7	50.7		67.4	26,469	45.8
(**b**)							
	**Resolution**	**I/O**	**RC**	**IF**	**SUM**	**Speedup**	**PSNR**
Arch. S1	256 × 256				1512.4	1	n/a
	512 × 512				5705.5	1	n/a
	1024 × 1024				22,473.4	1	n/a
	2048 × 2048				89,143.7	1	n/a
	4096 × 4096				355,409.1	1	n/a
Arch. S2	256 × 256	3.2	33.8	0.9	37.9	40	42.2
	512 × 512	3.2	34.0	1.4	38.6	148	45.3
	1024 × 1024	3.1	33.6	3.6	40.3	558	45.6
	2048 × 2048	3.3	33.9	11.5	48.7	1830	45.8
	4096 × 4096	3.1	33.8	46.3	83.3	4269	45.8
Arch. S3	256 × 256	3.4	2.4	0.9	6.8	222	42.2
	512 × 512	3.3	2.3	1.6	7.2	795	45.3
	1024 × 1024	3.2	2.0	3.9	9.1	2462	45.6
	2048 × 2048	3.2	2.4	11.7	17.2	5180	45.8
	4096 × 4096	3.3	2.4	46.9	52.7	6747	45.8
Arch. S3 (Multi-Stream)	256 × 256	3.4	2.5		5.9	256	42.2
	512 × 512	3.4	3.0		6.4	894	45.3
	1024 × 1024	3.4	3.2		6.6	3390	45.6
	2048 × 2048	3.4	10.3		13.7	6529	45.8
	4096 × 4096	3.4	40.1		43.5	8172	45.8
Arch. S3 (Multi-GPU)	256 × 256	3.2	6.0		9.3	163	42.2
	512 × 512	3.3	6.2		9.4	605	45.3
	1024 × 1024	3.2	6.2		9.4	2396	45.6
	2048 × 2048	3.2	6.6		9.7	9181	45.8
	4096 × 4096	3.2	10.4		13.6	26,152	45.8

**Table 8:** BP-VISAR frame generation time with the number of streams in the multi-stream based processing architecture, overlapped mode (α=0.8, unit: ms).

Resolution	Number of Streams							
	1-Way	2-Way	3-Way	4-Way	5-Way	6-Way	7-Way	8-Way
1024 × 1024	6.1	3.2	3.2	3.0	3.0	3.0	3.1	3.1
2048 × 2048	14.4	10.3	10.2	10.2	10.2	10.2	10.2	10.2
4096 × 4096	49.0	45.1	41.3	41.0	41.0	41.1	41.2	41.2

**Table 9:** Performance comparison with previous studies.

	[20]	[23]	Arch. F3		Arch. S3			
**SAR Mode**	**Image**	**Image**	**Video**		**Video**			
**Pixel-Resolution**	**4096** × **4096**	**8965** × **7065**	**4096** × **4096**		**4096** × **4096**			
aperture proc.	full	full	full	full	sub	sub	sub	sub
overlap ratio(%)	0	0	0	80	0	80	80	80
Np	300,000	8192	585	585	585	585	585	585
#GPU	1	4	1	1	1	1	1	4
#stream/GPU	n/a	n/a	1	1	1	1	3	3
#CUDA cores	10,496	8192	10,496	10,496	10,496	10,496	10,496	41,984
imaging time (s)	180.000	26.100	0.236	0.212	0.227	0.053	0.044	0.014
GPU clock(GHz)	1.70	1.17	1.70	1.70	1.70	1.70	1.70	1.70
normalized time	1.49	1.13	1.00	0.90	0.97	0.22	0.18	0.23

## Data Availability

Not applicable.

## Abbreviations

The following abbreviations are used in this manuscript:

BP-VISAR	Backprojection based Video SAR
BPA	Backprojection Algorithm
CBP	Convolution Backprojection
D2H	Device-to-Host
DS	Digital Spotlighting
FBP	Fast Backprojection
FPGA	Field-Programmable Gate Arrays
GPGPU	General-Purpose Graphics Processing Unit
H2D	Host-to-Device
IF	Image Formation
SM	Streaming Multiprocessor
PP	Post-Processing
RC	Range Compression
ROI	Region of Interest
SAR	Synthetic Aperture Radar
SFB	Sub-aperture Frame Buffer
SRPB	Sub-aperture Range Profile Buffer
